# President's Address

**Published:** 1907-06-15

**Authors:** 


### REPORT OF OHIO STATE DENTAL SOCIETY MEETING.

Meeting held December 4th, 5th and 6th, Columbus, O.

### President's Address.

President H. L. Ambler's address was on the topic of the value of helpfulness of the Dental Society. A dentist may never know how capable or useful he may make himself until he begins to associate with his confreres, and to labor for and with them for the advancement of their common cause. Attendance upon dental conventions then becomes a duty and when cordially entered upon one of the greatest pleasures a professional man can have. Every paper one hears read and discussed is an inspiration to one to study and be prepared to write himself, and every clinic one witnesses is like a challenge to one's pride that he may equal or excel it. As dentists, we don't need to visit foreign countries to learn our art; the best known to men is presented through our journals and societies, and we have only to attend these meetings and read our own journals to keep in touch with every advance made.

It is therefore incumbent on every conscientious practitioner to attend and participate in his local or state societies that he may serve his patients with that skill which is expected by them. He cited as an instance of devotion to his profession the fact that Dr. Jonathan Taft gave fully one-twelfth of his time to the public work of his profession. He succeeded and was honored among men, while other men of equal ability stayed at home and complained instead of lending a helping hand, and have died and never will be missed

Some men attend society meetings only for what they can absorb, and never assist in making the meetings more or less interesting or valuable by their participation in any way; but if they only knew it they are the very men who get the least out of the meetings. It is the man who puts himself *en raporte* with every part of the meeting that gets the most out of it. Petty jealousies or criticism of the management have spoiled many a man's pleasure and profit, and even destroyed the intended good influence of many a good meeting. Some of the advantages one gets are: Growth in professional knowledge and skill, formation and renewal of friendships, development of grander ideals, resolutions for better service to our patients, to say nothing of the recreation a few days out of the routine of office experience gives one. It is certainly safe to say that all the people know more than any one, and it is therefore good that we all get together in convention and relate what we think and know and can demonstrate.

DISCUSSION.

DR. H. A. SMITH, CINCINNATI: As I sat here listening my mind reverted to the day when I first joined a dental society. It was the day following my graduation, and the society was the old Mississippi Valley Association. Until it died I remained constantly a member. I have been a member of various other societies, and have been greatly benefited by association with their membership. I have frequently said that if the advantages of society associations had been omitted from the privileges which, as a dentist, I have so long enjoyed, not much would have been left for me. Lord Bacon said, "Every man is a debtor to his profession." I know that I have been profited more than can be estimated by association with members of the dental profession, enjoying not only the social features of such association, but gaining thereby an introduction to all that is best in the literature and the traditions of the profession.

Dentistry as a profession is young, not yet seventy years of age. Three factors have contributed to its elevation; dental literature, dental colleges, dental associations. Without these three agencies dentistry would be today merely a crude art. We cannot afford to omit any one of them. They have been the great fundamental influences which have developed our profession to its present proportions. As to the dental society, this Ohio State Society, the surprising fact is, that so comparatively few of the dentists avail themselves of its proffered benefits. Of the four hundred dentists of my own city, there are probably not more than fifty in this society. Of the large number of dentists in the entire state, a small proportion only attend. I cannot understand why this is so. Seeing my old friend, Dr. Beauman here, reminds me that he and I are the only surviving ones of the first membership of this society. I feel a thrill of pride that he still comes to aid the younger members of the society.

